# Chimeric Antigen Receptor-Redirected Regulatory T Cells Suppress Experimental Allergic Airway Inflammation, a Model of Asthma

**DOI:** 10.3389/fimmu.2017.01125

**Published:** 2017-09-12

**Authors:** Jelena Skuljec, Markus Chmielewski, Christine Happle, Anika Habener, Mandy Busse, Hinrich Abken, Gesine Hansen

**Affiliations:** ^1^Pediatric Pneumology, Allergology and Neonatology, Hannover Medical School, Hannover, Germany; ^2^Biomedical Research in Endstage and Obstructive Lung Disease Hannover (BREATH), German Center for Lung Research (DZL), Hannover, Germany; ^3^Center for Molecular Medicine Cologne, University of Cologne, Cologne, Germany; ^4^Clinic I Internal Medicine, University Hospital Cologne, Cologne, Germany

**Keywords:** allergic asthma, chimeric antigen receptor, regulatory T cells, adoptive cell therapy, ovalbumin mouse model

## Abstract

Cellular therapy with chimeric antigen receptor (CAR)-redirected cytotoxic T cells has shown impressive efficacy in the treatment of hematologic malignancies. We explored a regulatory T cell (Treg)-based therapy in the treatment of allergic airway inflammation, a model for asthma, which is characterized by an airway hyper-reactivity (AHR) and a chronic, T helper-2 (Th2) cell-dominated immune response to allergen. To restore the immune balance in the lung, we redirected Tregs by a CAR toward lung epithelia in mice upon experimentally induced allergic asthma, closely mimicking the clinical situation. Adoptively transferred CAR Tregs accumulated in the lung and in tracheobronchial lymph nodes, reduced AHR and diminished eosinophilic airway inflammation, indicated by lower cell numbers in the bronchoalveolar lavage fluid and decreased cell infiltrates in the lung. CAR Treg cells furthermore prevented excessive pulmonary mucus production as well as increase in allergen-specific IgE and Th2 cytokine levels in exposed animals. CAR Tregs were more efficient in controlling asthma than non-modified Tregs, indicating the pivotal role of specific Treg cell activation in the affected organ. Data demonstrate that lung targeting CAR Treg cells ameliorate key features of experimental airway inflammation, paving the way for cell therapy of severe allergic asthma.

## Introduction

Asthma is a very common chronic respiratory disease which affects more than 300 million people worldwide (1). It is characterized by airway inflammation, airway hyper-reactivity (AHR), and reversible airway obstruction. Asthma covers several distinct clinical and biological phenotypes whereof allergic asthma is a leading subtype (2). Treatment with anti-inflammatory and broncho-spasmolytic drugs succeeds to control symptoms in most patients (3), however, leaving 10–20% of patients refractory to any therapy (4). Apart from symptomatic treatment no curative therapy is available, which makes new approaches mandatory, especially for patients with severe and uncontrolled asthma.

Allergic asthma is associated with an overwhelming T helper-2 (Th2) cell-dominated immune response to allergens. Physiologically, airway inflammation is counteracted by inhibitory molecules and suppressor cells including CD4^+^ regulatory T cells (Tregs) (5, 6) which becomes visible upon Treg depletion which causes spontaneous asthma-like airway pathology (7). Patients suffering from allergic asthma have reduced numbers of Tregs that are furthermore impaired in their suppressive capacity (8–11). Some currently applied therapies aim at enhancing Treg cell number and function (8, 12), whereas adoptive transfer of Tregs can suppress both the priming and the effector phase of allergic airway inflammation in experimental models of murine asthma (13–15).

Antigen-specific Tregs seem to be superior in ameliorating an inflammatory disease compared with polyclonal Tregs (16–18). However, the translation to clinical application is limited by the very small number of antigen-specific Tregs in the peripheral blood which can be identified, isolated, and amplified for therapeutic purposes. The tremendous diversity of asthma eliciting antigens makes the identification of such specific Tregs extremely laborious with the risk that no specific cell clone can be identified. To provide pre-defined specificity, Tregs can be engineered *ex vivo* with a chimeric antigen receptor (CAR) (19) that harbors an antibody-derived binding domain for antigen and activates the engineered T cell upon binding independently of MHC recognition (20). While CAR engineered pro-inflammatory T cells are extensively studied and produced impressive efficacy in the treatment of hematologic malignancies in early phase trials (21), CAR engineered Tregs were only sporadically evaluated in animal models, e.g., for the treatment of colitis (22) and multiple sclerosis (23).

In this study, we assessed whether antigen-specifically redirected Tregs are capable to control experimentally induced airway inflammation in a clinically relevant mouse model mimicking the human situation. In order to target to the lung and to initiate Treg cell activation in the targeted tissue, we redirected Tregs by a CAR which recognizes carcinoembryonic antigen (CEA), a glycoprotein present on the surface of adenoepithelia in the lung and gastrointestinal tract. We demonstrate that CAR Tregs accumulate and are activated in the inflamed lung of asthmatic CEA transgenic (CEAtg) mice where they control key symptoms of allergic inflammation more efficiently than non-modified Tregs. The results imply a Treg cell-based strategy for the treatment of patients with severe allergic asthma.

## Materials and Methods

### Mice

Carcinoembryonic antigen transgenic C57BL/6 mice were obtained from the Patterson Institute, Manchester, UK. The CEAtg mouse colony was bred by back-crossing with a colony of C57BL/6N mice (Charles River, Wilmington, MA, USA). Offspring mice were genotyped using the primer oligonucleotides 5′-CTGCAGCTGTCCAATGGC-3′ and 5′-CCTGGGACTGACCGGGAG-3′. C57BL/6 wild-type (wt) mice were used as controls. CEA-specific CAR transgenic (CEA-CARtg) C57BL/6 mice were generated by the laboratory of Prof. Abken (unpublished data). Briefly, embryonic stem cells were transfected with the Cre/loxP rosa26 vector containing a CD4 promoter-driven expression cassette coding for the fully murine SCA431scFv-mIgG-CD4(tm)-CD28–CD3ζ CAR. Blood T cells express the CEA-specific CAR on the cell surface. All experimental protocols were approved by the local animal welfare committee Agency for Nature, Environment and Consumer Protection of the State North Rhine-Westphalia (LANUV) and performed according to their guidelines.

### Cell Isolation and Flow Cytometry Analysis

Single cell suspensions from spleens and tracheobronchial lymph nodes (LNs) were obtained by meshing organs through a 70 µm cell strainer (BD, Franklin Lakes, NJ, USA), followed by lysis of erythrocytes and filtering through 30 µm cell strainer (Miltenyi Biotec, Bergisch Gladbach, Germany). Cells were stained with the anti-mouse monoclonal antibodies (mAb) CD4-FITC/clone GK1.5 (Southern Biotech, Birmingham, AL, USA), CD25-PerCP-Cy5.5/clone PC61 (BD), PE-conjugated anti-mouse CD25 mAb clone 7D4 (Miltenyi), FITC-labeled anti-mouse CD4 mAb clone GK1.5 (Southern Biotec), AF488-labeled anti-FoxP3/clone MF-14 (BioLegend, San Diego, CA, USA), RPE-conjugated anti-IgG_1_ that binds to the CAR extracellular Fc domain (Southern Biotech), anti-latency-associated peptide (LAP)/clone TW7-16B4 (BioLegend), and “FoxP3 Staining Buffer Set” (Miltenyi Biotec). Data were acquired on FACS Canto II flow cytometer (BD) and analyzed using FlowJo software (FlowJo, LCC, Ashland, OR, USA).

### *In Vitro* Assays

T effector cells (Teffs) and Tregs were isolated from the murine spleens using the “Pan T Cell Isolation Kit II” and the “CD4^+^CD25^+^ Regulatory T Cell Isolation Kit” (Miltenyi Biotec), respectively, or using the “autoMACS” (Miltenyi Biotec). CAR Teffs were stained with PKH26 (Sigma-Aldrich, St. Louis, MO, USA) and stimulated for proliferation either by the immobilized agonistic mAb anti-CD3/clone 145-2C11 and anti-CD28/clone 37.51, or the anti-idiotypic CAR-activating mAb BW2064/36 directed against the IgG_1_ spacer domain of the CAR (5 µg/ml coating concentration each). Teffs (10^5^ cells/well) were incubated with or without CAR Tregs (5 × 10^4^ cells/well) for 48 h. Intensity of PKH26 dye was recorded by flow cytometry using FACS Canto II and the proliferation rate of Teffs was calculated. For the LAP expression analysis, Treg cells were stimulated by incubation on plates coated with the anti-CD3 and anti-CD28 mAb, whereas anti-CEA CAR Tregs were incubated on plates coated with the BW2064/36 (anti-CAR mAb) for 48 h (5 × 10^4^ cells/well). Mock-coated plates (w/o) were used for control. LAP on the surface was detected using LAP-specific mAb using FACS Canto II. For IL-10 expression assay, CAR Tregs were cultured in triplicates in microtiter plates (PolySorp; Nalge Nunc, Rochester, NY, USA) precoated with the BW2064/36 mAb or an IgG_1_ control mAb (Southern Biotech). After 72 h, IL-10 was measured in culture supernatants with the “mTh1/Th2/Th9/Th17/Th22/Treg Cytokine Panel 17-plex” (eBioscience/Thermo Fisher Scientific, Waltham, USA).

### *In Vivo* Cell Tracking with Bioluminescence Imaging

CAR Tregs and non-modified (wt) Tregs were retrovirally transduced to express Gaussia Luciferase (GLuc) and intravenously (i.v.) injected to ovalbumin (OVA)-sensitized CEAtg mice (24). 36 h later, Gluc-labeled cells were visualized by intraperitoneal (i.p.) injection of 100 µg benzyl-coelenterazine (PJK GmbH, Kleinblittersdorf, Germany). Lungs, spleen, stomach, and kidney were isolated and in the Petri dish recorded with 300 s exposure time using the Photon Imager (Biospace Lab, Nesles-la-Vallée, France). The threshold of bioluminescence signals was automatically determined using the Photo Vision software (Biospace Lab) and filtered against the background noise. Regions of interest were defined as regions above threshold and automatically gated by pre-defined program tools.

### Induction of Allergic Airway Inflammation and Adoptive Transfer of Tregs

Ovalbumin (Grade V, Sigma-Aldrich) was used as model allergen (25). Lipopolysaccharide was removed from OVA by the Detoxi-Gel endotoxin removing gel and columns (Thermo Fisher Scientific). Clearance from endotoxin was confirmed by the Limulus amebocyte lysate test (Lonza, Basel, Switzerland). CEAtg mice were sensitized with two i.p. applications of 20 µg OVA in saline solution, adsorbed to 2 mg of aluminum hydroxide and magnesium hydroxide solution (alum; Inject alum adjuvant, Thermo Fisher Scientific), followed by intranasal (i.n.) exposures with 20 µg OVA at four consecutive days. Control mice were likewise treated with solutions without OVA. CAR Tregs for adoptive transfer were isolated from the spleens of the CEA-CARtg mouse or the C57BL/6 wt mouse using the “CD4^+^CD25^+^ Regulatory T cell Isolation Kit” (Miltenyi Biotec). Seven days after the first sensitization with allergen, CAR Tregs or wt Tregs were i.v. injected (1 × 10^6^ cells per mouse). One day after the last i.n. challenge, mice were sacrificed by asphyxiation with isoflurane (Baxter, Deerfield, IL, USA).

### Measurement of AHR

Airway hyper-reactivity was defined as an increase in dynamic lung resistance (R) in response to β-methacholine (MeCh; Sigma-Aldrich). One day after the last challenge with allergen, mice were deeply anesthetized with ketamine (Albrecht GmbH, Aulendorf, Germany) and xylazine (Rompun, Bayer Vital GmbH, Leverkusen, Germany), intubated, and mechanically ventilated *via* the flexiVent system (Scireq, Montreal, QC, Canada) using a tidal volume of 12 ml/kg at a frequency of 150 breaths/min. For baseline measurements mice were exposed to 0.9% (w/v) NaCl, aerosolized in the Aeroneb nebulizer (Inspiration Medical, Bochum, Germany). Subsequently, mice were provoked with increasing concentrations of MeCh (10, 20, and 30 mg/ml). After deep inflation of the lungs, single-frequency forced oscillations were performed; four peak values of R were analyzed for each MeCh concentration. AHR data were calculated as the change from baseline response.

### Histological Stainings

Cryostat sections were stained with the biotin-conjugated anti-CEA mAb clone CB30 (Ancell, Bayport, MN, USA), streptavidin-horseradish peroxidase (BioLegend), and DAB chromogen substrate (Biozol, Eching, Germany). Sections were additionally stained with hematoxylin–eosin (H&E; Carl Roth, Karlsruhe, Germany) and analyzed using the Axiovert 400 M laser scan microscope (Carl Zeiss, Oberkochen, Germany). The left lung was fixed in 4% (w/v) formalin and embedded in paraffin. Tissue slices were stained with H&E (Merck, Darmstadt, Germany) or periodic acid-Schiff reagent (PAS; Sigma-Aldrich). Whole lung sections were scanned using the Keyence microscope BZ-9000 (Keyence, Osaka, Japan) at 100× magnification. Sum of H&E- or PAS-positive signal (pixels) per lung slice was quantified using in-house developed imaging software (25). For the immuno-histological detection of Tregs, lung and spleen cells were embedded in “Tissue-Tek O.C.T. Compound” (Sakura Finetek Europe B.V., Alphen aan den Rijn, Netherlands) and 5-µm cryostat sections were fixed in ice-cold acetone. Sections were stained for CD4 and FoxP3 expression with the fluorochrome-conjugated antibodies specific for mouse CD4-AF594/clone GKL1.5, FoxP3-AF488/clone 150D (BioLegend), and DAPI (4,6-diamidino-2-phenylindole dihydrochloride) for nuclear counterstain. For the detection of the CAR expression on the surface of Tregs, sections were incubated with the hybridoma-derived anti-idiotypic antibody BW2064/36 previously biotinylated by the Sulfo-NHS-LC-Biotin kit (Thermo Fisher Scientific). Following incubation with the primary antibody, antibody binding was visualized using AF-647 labeled streptavidin (BioLegend). Magnification was set to 60×. Slides were analyzed using the Olympus FV 1000 microscope (Olympus corporation, Tokio, Japan).

### Analysis of Bronchoalveolar Lavage Fluid (BALF)

To obtain BALF, the right lung was flushed three times with 0.4 ml 2 mM EDTA in PBS. The total cell number was determined using the Cedex HiRes automated cell analyzer (Roche, Basel, Switzerland). To determine the cell types, cytospin slides were made with the CytoSpin 4 Cytocentrifuge (Thermo Fisher Scientific) and stained with May-Grünwald/Giemsa (Merck). Cell types were determined using standard microscopic criteria and counted in a blinded manner at 1000× magnification (Axiovert 40 CFL, Zeiss, Oberkochen, Germany).

### Quantification of OVA-Specific Immunoglobulins (Ig) and Cytokines

Peripheral blood was clotted at room temperature for 20 min and the serum was collected. OVA-specific IgE, IgG_1_, and IgG_2a_ were recorded by ELISA. Serum samples were incubated overnight in microtiter plates (Nalge Nunc) that were previously coated with 10 µg/ml OVA in bicarbonate buffer (pH 9.6). Specific binding was detected with respective goat anti-mouse horseradish peroxidase-conjugated anti-IgE antibodies (Southern Biotech), anti-IgG_1_, or anti-IgG_2a_ antibodies (Bethyl Laboratories, Inc., Montgomery, TX, USA) and 3,3′,5,5′-tetramethylbenzidine as a colorimetric substrate. Optical density was determined at 450/630 nm using a microplate reader (Bio-Rad Laboratories, Inc., Hercules, CA, USA). OVA-specific IgE concentration in samples was determined using a standard calibration curve (AbD Serotec, Kidlington, UK). The titers of OVA-specific IgG_1_ and IgG_2a_ were calculated by logarithmic regression as the reciprocal dilution of the sera. Cytokines in sera or cell culture supernatants were measured using the bead-based “Mouse 17-plex Bio-Plex multiplex system” and the Luminex xMAP device (Bio-Rad) or FlowCytomix (eBioscience/Thermo Fisher Scientific) and FACS Canto II.

### Gene Expression Analysis

The presence of CAR Tregs in tissues after adoptive transfer was additionally analyzed by RT-PCR. RNA was isolated from 4 to 5 million cells with “RNeasy Mini Kit” and “RNase-Free DNase Set” (Qiagen, Venlo, Netherlands). CAR cells were detected using OneStep RT-PCR Kit (Qiagen) and CAR specific primer oligonucleotides (5′-AAACAAACTGGAATGGATGGGCTACA-3′ and 5′-AACGTGGGATAACTACTCCACTGAT-3′).

### Statistical Analysis

Unpaired two-tailed Student’s *t*-test, one-way, or two-way ANOVA test with Bonferroni’s multiple comparison post-test (95% confidence interval) was performed using GraphPad Prism (San Diego, CA, USA). All data represent the mean ± SEM. Differences were considered statistically significant at *p* < 0.05 (*), *p* < 0.01 (**), and *p* < 0.001 (***).

## Results

### CAR Tregs Suppress the Proliferation of Teff Cells

**Figure 1:**
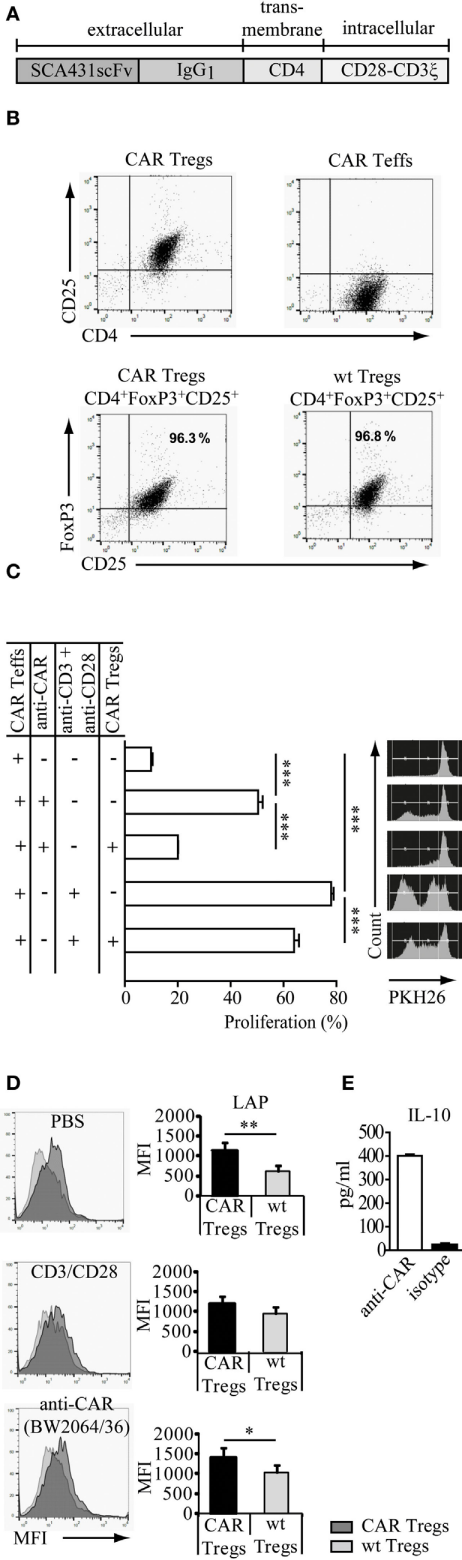
Regulatory T cells (Tregs) engineered with a carcinoembryonic antigen (CEA)-specific chimeric antigen receptor (CAR) specifically redirect their suppressive capacity. **(A)** Modular composition of the CEA-specific CAR with the anti-CEA scFv binding domain and the combined CD28–CD3ζ signaling domains. **(B)** CD4^+^CD25^+^ Tregs and CD4^+^CD25^−^ T effector cells (Teffs) with anti-CEA CAR were isolated from the spleens of the anti-CEA CAR transgenic mice. Tregs isolated from CEA transgenic mice or wild-type (wt) C57BL/6 mice stained positive for CD4, CD25, and FoxP3. **(C)** CAR Tregs suppressed the amplification of PKH26-labeled CAR Teffs that were stimulated by the agonistic anti-CD3/anti-CD28 antibodies directed against the TCR/CD28 or by the BW2064/26 antibody (anti-CAR) directed against the CAR. Samples were measured in triplicates. Statistical analyses were performed by the one-way ANOVA test. ***p < 0.001. **(D)** Latency-associated peptide (LAP) (TGF-β1) is increased on the surface of CAR engineered CD4^+^CD25^+^FoxP3^+^ Treg cells upon specific stimulation. The assay was performed in triplicates. Statistical analyses were performed by the Student’s *t*-test (*p < 0.05, **p < 0.01). **(E)** CAR Tregs were stimulated by the BW2064/36 monoclonal antibodies (mAb) (anti-CAR) as surrogate antigen or by an IgG_1_ isotype matched control mAb. IL-10 released into the supernatant was recorded by a bead-based immunoassay. Data are representative for two independent experiments.

The anti-CEA CAR was composed of the anti-CEA single chain variable fragment (scFv) for binding, the IgG_1_ hinge-CH2CH3 as an extracellular spacer, the CD4 trans-membrane domain, and the intracellular CD28 and CD3ζ signaling domains for T cell activation upon binding to CEA (Figure 1A). CD4^+^CD25^+^ Tregs and CD4^+^CD25^−^ Teffs with anti-CEA CAR were isolated from the spleens of the anti-CEA CARtg mice. CAR Tregs stained positive for CD4, CD25, high level FoxP3 (Figure 1B), and the engineered CAR (not shown). To demonstrate the suppressive activity of CD4^+^CD25^+^ Tregs, cells were co-cultured with PKH26-labeled CD3^+^CD25^low^ CAR Teffs. CAR Tregs effectively repressed the proliferation of CD3^+^CD25^low^ CAR Teffs when stimulated through TCR/CD28 by the agonistic anti-CD3 and anti-CD28 mAb or through the CAR by the CAR-binding antibody BW2064/26 (Figure 1C). We conclude that the CD4^+^CD25^+^ CAR Treg cells constitute functionally active Tregs capable to suppress CAR Teff amplification. Treg cells, stimulated through the CAR, suppressed Teff cell proliferation more efficiently than after stimulation through CD3/CD28. This is likely due to differences in the CAR versus CD3/TCR-mediated T cell activation, i.e., the CD28ζ CAR provided much stronger CD28/CD3ζ signals than CD3/CD28 stimulation. Since the activation of Treg cells depends on the strength of the CD28 stimulation (19), stimulation through the CD28–CD3ζ CAR is likely superior to CD3/TCR stimulation. In accordance to our conclusion, CAR Treg cells showed higher activation levels than non-modified, wt Treg cells indicated by higher levels of LAP (TGF-β1) expression, as revealed by flow cytometry (Figure 1D). Furthermore, IL-10 production by CAR Tregs increased upon CAR stimulation through a specific antibody compared with the incubation with an isotype antibody of irrelevant specificity as control (Figure 1E). We concluded that antigen engagement by the CAR improved Treg cell activation in a specific fashion.

### Anti-CEA CAR Tregs Home to the Lung of CEAtg Mice

**Figure 2:**
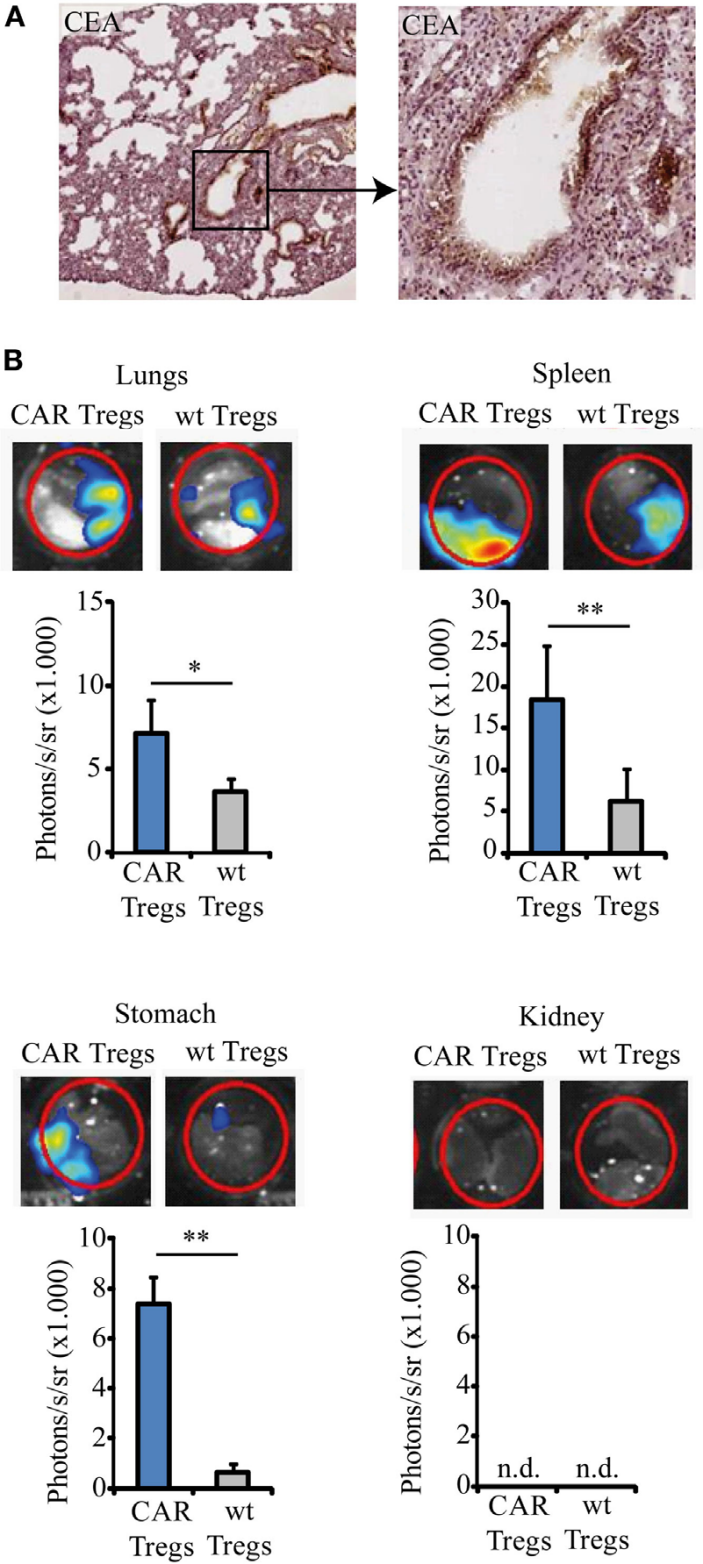
Chimeric antigen receptor (CAR) regulatory T cells (Tregs) home to the lung and spleen. **(A)** Lung slices from the carcinoembryonic antigen transgenic (CEAtg) mouse were stained for CEA (microscope magnifications 5× and 20×, respectively). CEA is expressed in a polarized fashion on the luminal site by the alveolar epithelia. **(B)** Gaussia Luciferase (Gluc)-labeled CAR Tregs and Gluc-labeled non-modified wild-type (wt) Tregs were recorded by bioluminescent imaging in the lungs, spleen, stomach, and kidney in the same mouse 36 h after Treg transfer to the ovalbumin-sensitized mice. One representative mouse out of three from each group is shown. Statistical analyses were performed using the Student’s *t*-test. *p < 0.05, **p < 0.01; n.d., not detectable.

We used the immunocompetent CEAtg mice as recipients to evaluate in a clinically relevant model the immune-modulating effect of adoptively transferred CEA-specific CAR Tregs during induced allergic airway inflammation. The CEAtg mice express CEA under the control of the human CEA promoter on the luminal surface of the pulmonary (Figure 2A) and the gastrointestinal tract epithelia, closely mimicking the human situation. These mice were pre-sensitized by exposure to OVA; subsequently, one dose of non-preactivated, Gluc-labeled anti-CEA CAR Tregs or non-modified Tregs were applied by i.v. injection. CAR Tregs accumulated in the CEA^+^ lung, spleen, and stomach of OVA-treated mice 36 h after i.v. application while wt Tregs without CAR did far less (Figure 2B). Accumulation in CEA^+^ organs is specific since CAR Tregs did not substantially infiltrate the CEA^−^ kidneys.

### CAR Tregs Prevent AHR, Eosinophilic Airway Inflammation, Mucus Production, and Th2 Cytokine Production in Mice with Experimental Asthma

**Figure 3:**
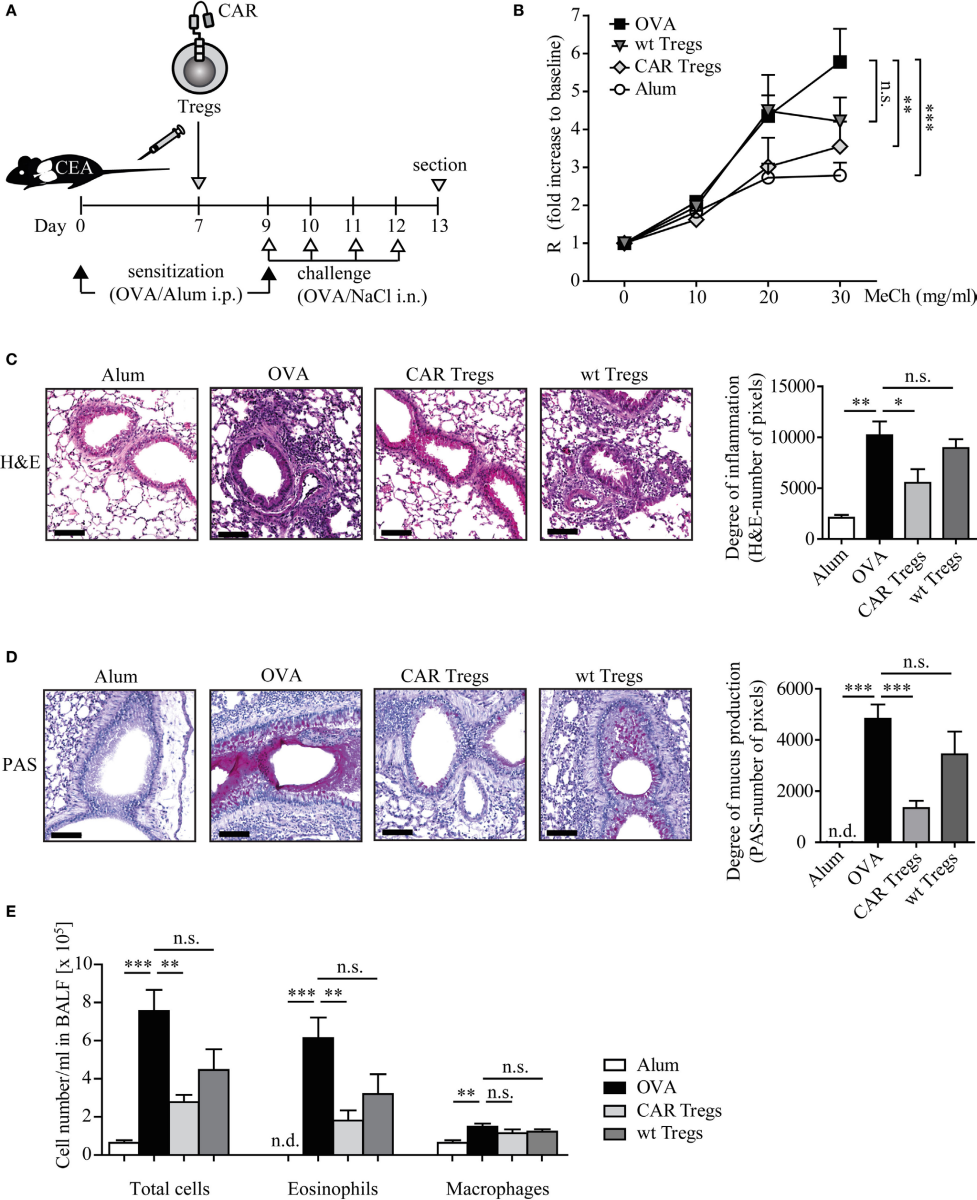
Adoptive transfer of chimeric antigen receptor (CAR) regulatory T cells (Tregs) reduces airway hyper-reactivity, inflammation, mucus production, and eosinophilia in mice with induced experimental asthma. **(A)** Schematic outline of the experimental protocol. **(B)** Lung resistance (R) after exposure to increasing concentrations of methacholine (MeCh) was recorded as described in Section “Materials and Methods.” Data from *n* = 9–11 mice from three independent experiments are shown. Statistical analyses were performed with the two-way ANOVA test. **(C,D)** Stained whole lung sections were quantified for **(C)** hematoxylin–eosin (H&E) or **(D)** PAS signals (number of pixels), *n* = 4–6 (H&E) and *n* = 6–9 (PAS) each from 3 independent experiments; scale bar = 100 μm. **(E)** Absolute numbers of cells in the bronchoalveolar lavage fluid (BALF); *n* = 5–7 in one of four independent experiments. Alum, mice treated with alum adjuvant only; ovalbumin (OVA), mice treated with OVA in alum adjuvant; CAR Tregs, mice treated with OVA in alum adjuvant and subsequent one dose of CAR Treg cells; wild-type (wt) Tregs, mice treated with OVA in alum adjuvant and subsequent one dose of unmodified wt Treg cells. Statistical analyses were performed by the one-way ANOVA test. *p < 0.05, **p < 0.01, ***p < 0.001; n.s., not significant, n.d., not detectable.

We investigated whether adoptive transfer of CAR-redirected Tregs can suppress the clinical symptoms of experimental asthma in the mouse model. By systemic sensitization and local challenges with the model allergen OVA (Figure 3A), we induced the typical key features of asthma, such as AHR, eosinophilic airway inflammation, increased Th2 cytokine production, and elevated serum IgE levels. Control animals, inoculated with alum or NaCl solution without OVA, did not show these symptoms.

Adoptive transfer of CAR Tregs by one dose of i.v. injection to OVA-challenged CEAtg mice almost completely prevented the development of MeCh-induced AHR, whereas unmodified Tregs were less efficient as determined by invasive lung function measurements (Figure 3B). Histological examination of the lung sections revealed a substantial reduction of infiltrating inflammatory immune cells in the CAR Treg-treated mice compared with the non-treated asthmatic mice (Figure 3C). Treatment with Tregs without CAR did not substantially reduce airway inflammation demonstrating the superior therapeutic efficacy of redirecting Tregs by a CAR toward the affected organ.

Likewise, mucus production, one of the major hallmarks of asthma, was significantly suppressed upon CAR Treg cell therapy as revealed by PAS staining of the lung tissue (Figure 3D). In this respect, Tregs without CAR showed no substantial therapeutic effect.

Adoptive transfer of CAR Tregs reduced the number of eosinophils in the BALF by about threefold as compared with mice without Treg treatment while the number of macrophages was not altered (Figure 3E). Mice which received unmodified Tregs showed a less pronounced decrease in the number of total BALF cells and particularly of eosinophils.

**Figure 4:**
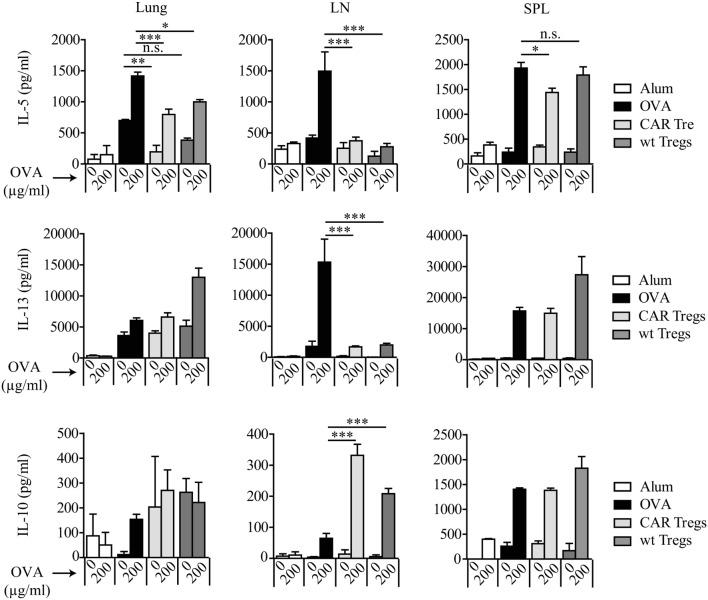
Chimeric antigen receptor (CAR) regulatory T cells (Tregs) reduce antigen-specific T helper-2 cytokine production by lung cells and splenocytes. IL-5, IL-13, and IL-10 levels were measured by bead-based assay in lung, spleen (SPL), and tracheobronchial lymph node (LN) cell culture supernatants after *in vitro* restimulation with 200 µg/ml ovalbumin (OVA). Data represent three independent experiments including pooled samples from more than 6 mice per group. Statistical analyses were performed by the one-way ANOVA test. *p < 0.05, **p < 0.01, ***p < 0.001.

Allergic asthma is characterized by a Th2-dominated immune response. To determine whether adoptive cell therapy with CAR Tregs modulates Th2 cytokine levels in OVA-challenged mice, we analyzed IL-5, IL-13, and IL-10 production, indicating progression of the disease. The superior suppression of asthma-like phenotype by CAR Treg treatment in relation to wt Treg injection was again demonstrated by significant reduction of antigen-specific IL-5 levels in cell culture supernatants of lung cells and splenocytes after *in vitro* restimulation with OVA (Figure 4).

### Adoptive Transfer of CAR Tregs Prevents the Allergen-Induced Increase of IL-5 and IgE

**Figure 5:**
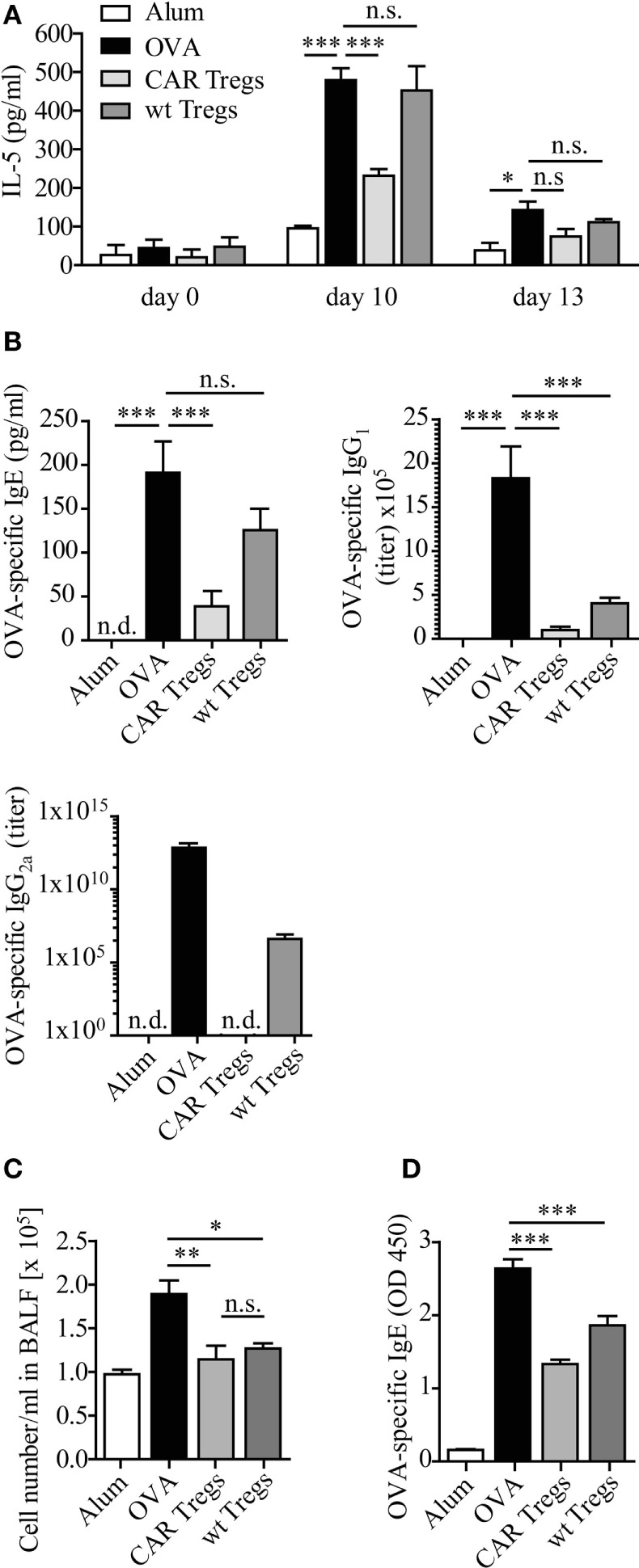
IL-5 and allergen-specific IgE levels are diminished upon chimeric antigen receptor (CAR) regulatory T cell (Treg) treatment of carcinoembryonic antigen transgenic (CEAtg) mice. **(A)** IL-5 levels in sera from CEAtg mice were determined at different times of the experimental protocol (Figure 3A), *n* = 3 mice per group per time point; statistical analysis was performed using the two-way ANOVA test. **(B)** The amount of ovalbumin (OVA)-specific IgE (*n* = 6–8 mice), IgG_1_ (*n* = 6–9), and IgG_2a_ (*n* = 6–17) in sera of CEAtg mice. Data are pooled from two to three independent experiments. **(C)** Total number of bronchoalveolar lavage fluid (BALF) cells in wild-type (wt) C57BL/6 mice without CEA expression, treated as described in Section “Materials and Methods.” **(D)** OVA-specific IgE at day 12 in sera of C57BL/6 mice was recorded by ELISA. Statistical analysis was performed by the one-way ANOVA test, *p < 0.05, **p < 0.01, ***p < 0.001.

We further examined IL-5 levels in mouse sera. CAR Treg-treated mice displayed significantly reduced IL-5 levels compared with untreated mice or mice treated with unmodified Tregs (Figure 5A). The effect was most prominent as early as 3 days after transfer of CAR Tregs, i.e., at day after second OVA inoculation.

OVA induced experimental asthma was accompanied by an increase of OVA-specific Ig levels in serum (Figure 5B). Adoptive CAR Treg transfer reduced the OVA-specific IgE levels, while non-modified Tregs did not. Essentially the same results were observed for IgG_2a_, while IgG_1_ levels were similarly reduced by Tregs with and without CAR (Figure 5B).

The pronounced effect of CAR Tregs compared with non-modified Tregs was due to CAR-mediated Treg cell activation. Transfer of CAR Tregs to OVA-treated wt C57BL/6 mice without CEA expression expectedly showed reduction in cell numbers in BALF compared to non-treated mice. However, the CAR Tregs were equally potent as the non-modified Tregs due to the lack of CAR-mediated Treg activation (Figure 5C). Accordingly, the levels of OVA-specific IgE were similarly reduced after Treg treatment with or without CAR (Figure 5D). We conclude that the suppressor activity of adoptively transferred Treg cells was substantially improved by anti-CEA CAR signaling through engagement of endogenous CEA.

### CAR Tregs Accumulate in the Lung, Spleen, and Regional LNs after Antigen Challenge

**Figure 6:**
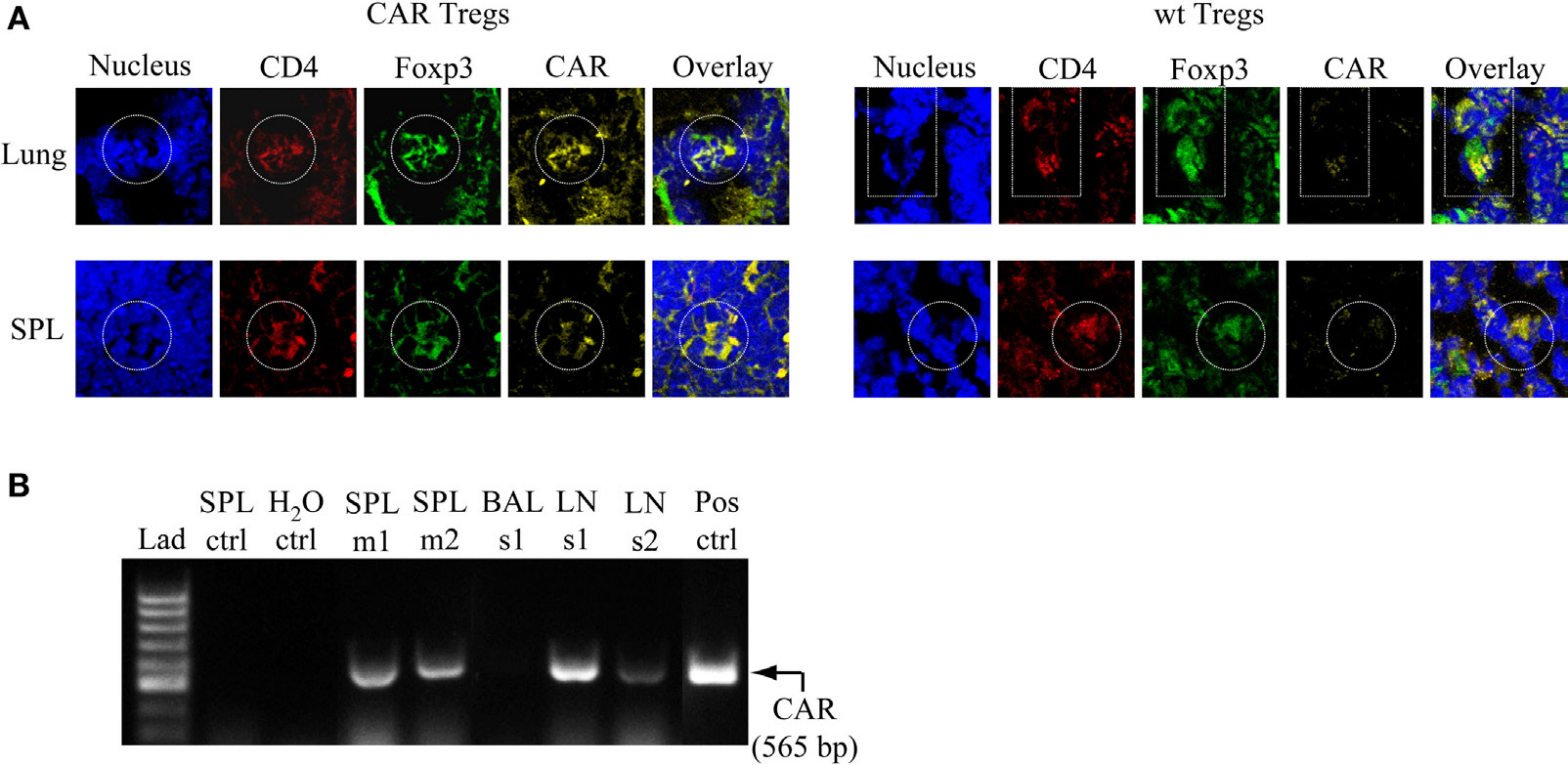
Chimeric antigen receptor (CAR) regulatory T cells (Tregs) accumulate in the lung, spleen (SPL), and tracheobronchial lymph nodes (LNs) after challenge with antigen. **(A)** Lung and SPL cells were isolated at day 13 from carcinoembryonic antigen transgenic mice treated with CAR Tregs or non-modified [wild-type (wt)] Tregs and fluorescently stained for nuclei (DAPI), and CD4, FoxP3, and CAR expression. One representative mouse out of three per group is shown. **(B)** Detection of CAR Tregs in the SPL and tracheobronchial LNs by RT-PCR at the end of experimental protocol (day 13). The specific RT-PCR CAR fragment is 565 bp in size. As expected, tissues from mice which received non-modified Tregs (SPL ctrl) did not show a CAR-derived signal. Bronchoalveolar lavage fluid (BALF) did not contain detectable CAR Treg cells. Lad, ladder of DNA fragments of different sizes; H_2_O ctrl, RT-PCR in the absence of RNA; Pos ctrl, RT-PCR with RNA from purified CAR T cells; m, mouse; s, sample consisting of cells that are pooled from five to six mice.

To examine the presence of CAR Tregs in CEAtg mice after the last antigen challenge at day 12, lung and spleen cells were fluorescently stained with specific anti-CD4, anti-CAR, and anti-FoxP3 antibodies. At this time point, CD4^+^CAR^+^FoxP3^+^ cells were still present in the lung and spleen of the CAR Treg treated animals, whereas tissues from mice treated with non-modified Tregs did not stain positive for the CAR signal, as expected (Figure 6A). RT-PCR analysis confirmed the immunostaining data and furthermore revealed that the CAR Tregs also accumulated in the tracheobronchial LNs (Figure 6B).

## Discussion

We show in an established mouse model that CAR-redirected Tregs effectively suppress the pathophysiological hallmarks of allergic airway inflammation. Targeted CD4^+^CD25^+^ Tregs significantly reduced AHR, airway eosinophilia, mucus hyper-secretion, Th2 cytokine production, and allergen-specific IgE after sensitization with a model allergen. CAR Tregs efficiently reduced proliferation of CAR Teffs *in vitro* after stimulation by their TCR or by the CAR through binding to cognate antigen. Concomitantly, LAP (TGF-1β) and IL-10 expression was increased after CAR activation. The CAR amplifies Treg cell function by providing strong activation through CD28–CD3ζ CAR signaling and by trapping the cells in the target tissue through binding to cognate antigen (20). At least two processes are taking place to improve CAR Treg function during chronic inflammation. (i) Airway inflammation results in some disruption of the lung epithelial layer with the consequence that those epithelial cells lose the luminal CEA expression and expose CEA also to the stromal site; by contrast, intact lung epithelia express CEA exclusively to the luminal site. Consequently, CAR Tregs in the inflamed lung are more strongly activated by CEA^+^ lung epithelia with de-polarized CEA expression while Tregs without CAR do not receive such activation signals. (ii) Tregs with anti-CEA CAR without engagement of cognate antigen are preactivated on a higher level, as indicated by increased LAP expression compared with Tregs without CAR (Figure 1D). This is likely due to the “tonus” of the anti-CEA CAR providing antigen-independent Treg cell activation. However, CEA engagement of cognate antigen further improved Treg cell activation, as indicated by a further increase in LAP. In contrast to CAR-redirected Tregs, the same number of unmodified Tregs did not show the striking therapeutic effect potentially due to the lack of activation. Once activated, Tregs suppress excessive immune responses in an antigen-independent fashion and can promote expansion of other Treg cells with different antigen specificities (26, 27). Our results are in line with the observation that preactivated Tregs, but less non-activated Tregs, suppress the airway inflammation in allergen-sensitized mice (28). The establishment of a protective Treg cell effect requires continuous antigen stimulation (29, 30). This is provided by the CEA^+^ lung epithelia in the CEAtg mouse model which is also expected to be the case in the human situation. In mice lacking transgenic CEA as source of continuous antigen stimulation, CAR Tregs were similarly efficient as non-modified Tregs in the suppression of inflammation and reduction of OVA-specific IgE levels (Figures 5C,D). Antigen-dependent Treg activation as trigger for the suppression of chronic inflammation was also reported for other disease models, such as multiple sclerosis (16), colitis (18), autoimmune diabetes (17), and gastritis (31).

The higher therapeutic efficacy of antigen-specific Tregs implies that a lower cell number is sufficient for the therapeutic effect than using polyclonal Tregs with diverse specificities. As previously shown, transfer of 5 × 10^6^ polyclonal, *in vitro* expanded CD4^+^CD25^+^ Tregs successfully suppressed airway inflammation in an allergic asthma model (14), whereas a 10-fold lower number of allergen-specific Treg cells were needed for the similar effect (13). In our study, one dose of 10^6^ CAR Tregs significantly ameliorated experimental asthma, and even lower cell numbers may be equally efficient.

Accumulation of Tregs at the site of inflammation is essential for their immunosuppressive function (32). In our model, redirecting of Tregs by an antigen-specific CAR sustained their recruitment to the targeted tissue. Accordingly, anti-CEA CAR Tregs accumulated in the CEA^+^ lung and spleen at higher levels than unmodified Tregs as revealed by bioluminescence monitoring. CAR Tregs, moreover, accumulated in the tracheobronchial LNs, lung, and spleen, even after repetitive challenge with antigen. Rapid and efficient trafficking to these tissues likely enables Tregs to modulate the ongoing chronic inflammation in the affected tissues.

T helper-2 cell-associated asthma arises from a complex interplay between innate and adaptive immune cells (2) and it is mediated by cytokines that induce AHR, recruitment of eosinophils and mast cells to the airways, and the production of allergen-specific IgE (33). Here, we show that CAR Tregs suppressed the production of IL-5 which is predominantly produced by Teffs (34) and which drives pulmonary eosinophilia and AHR (35). Accordingly, airway inflammation, eosinophilia, and AHR were suppressed upon the application of CAR Tregs. Suppression by Tregs in the allergic asthma model is likely mediated by IL-10 released by activated Tregs in high levels (13); other mechanisms may likewise apply such as induced cell death of responder T cells (36) and inhibition of T cell proliferation by upregulating cyclic adenosine monophosphate (37) or by CD39- and CD73-dependent peri-cellular generation of adenosine (38). The suppression of airway inflammation by Tregs may be due to a combination of these and other suppressive mechanisms (27, 39).

While the data indicate that genetically redirected Tregs are of therapeutic benefit in the treatment of severe asthma, the choice of the targeted antigen to redirect and activate Treg cells remains crucial. One option to redirect Tregs may be targeting of the specific allergen by the engineered CAR which, however, is frequently not known. Alternatively, CARs may be used to activate Tregs at the site of inflammation independently of the specific allergen in order to redirect their suppressive activity to the diseased lesion.

Cell therapy with Tregs has lately been tested in the treatment of refractory autoimmune diseases and high-risk allograft recipients (40). In this context, the stability of the Treg cell suppressive phenotype is a major concern. In particular, in the inflammatory environment Tregs can convert into Th17 cells (41) which could furthermore exacerbate the asthma symptoms (42). Several options were proposed to improve survival and function, including co-administration of IL-2 (43), all-trans retinoic acid (44), rapamycin (45), or ectopic FoxP3 expression in Tregs (46).

In our experimental model, adoptively transferred CAR Tregs preferentially accumulate in the lung, tracheobronchial LNs, and spleen where they efficiently suppress airway inflammation and other hallmarks of allergic asthma. Our results encourage further studies to explore adoptive cell therapy with genetically redirected Tregs as an efficacious treatment option for patients suffering from severe asthma.

## Ethics Statement

All experimental protocols were approved by the local animal welfare committee Agency for Nature, Environment and Consumer Protection of the State North Rhine-Westphalia (LANUV) and performed according to their guidelines.

## Author Contributions

JS, MC, HA, and GH designed the work. JS, MC, CH, AH, and MB acquired and analyzed experimental data. JS, MC, CH, AH, HA, and GH interpreted data. JS, MC, HA, and GH drafted the manuscript. All authors critically revised the work. JS, MC, HA, and GH contributed equally to this work.

## Conflict of Interest Statement

The authors declare that the research was conducted in the absence of any commercial or financial relationships that could be construed as a potential conflict of interest.
